# Feasibility of investigating differential proteomic expression in depression: implications for biomarker development in mood disorders

**DOI:** 10.1038/tp.2015.185

**Published:** 2015-12-08

**Authors:** M A Frye, M Nassan, G D Jenkins, S Kung, M Veldic, B A Palmer, S E Feeder, S J Tye, D S Choi, J M Biernacka

**Affiliations:** 1 Department of Psychiatry & Psychology, Mayo Clinic Depression Center, Mayo Clinic, Rochester, MN, USA; 2 Department of Health Sciences Research, Mayo Clinic, Rochester, MN, USA; 3 Department of Molecular Pharmacology & Experimental Therapeutics, Mayo Clinic, Rochester, MN, USA

## Abstract

The objective of this study was to determine whether proteomic profiling in serum samples can be utilized in identifying and differentiating mood disorders. A consecutive sample of patients with a confirmed diagnosis of unipolar (UP *n*=52) or bipolar depression (BP-I *n*=46, BP-II *n*=49) and controls (*n*=141) were recruited. A 7.5-ml blood sample was drawn for proteomic multiplex profiling of 320 proteins utilizing the Myriad RBM Discovery Multi-Analyte Profiling platform. After correcting for multiple testing and adjusting for covariates, growth differentiation factor 15 (GDF-15), hemopexin (HPX), hepsin (HPN), matrix metalloproteinase-7 (MMP-7), retinol-binding protein 4 (RBP-4) and transthyretin (TTR) all showed statistically significant differences among groups. In a series of three *post hoc* analyses correcting for multiple testing, MMP-7 was significantly different in mood disorder (BP-I+BP-II+UP) vs controls, MMP-7, GDF-15, HPN were significantly different in bipolar cases (BP-I+BP-II) vs controls, and GDF-15, HPX, HPN, RBP-4 and TTR proteins were all significantly different in BP-I vs controls. Good diagnostic accuracy (ROC-AUC⩾0.8) was obtained most notably for GDF-15, RBP-4 and TTR when comparing BP-I vs controls. While based on a small sample not adjusted for medication state, this discovery sample with a conservative method of correction suggests feasibility in using proteomic panels to assist in identifying and distinguishing mood disorders, in particular bipolar I disorder. Replication studies for confirmation, consideration of state vs trait serial assays to delineate proteomic expression of bipolar depression vs previous mania, and utility studies to assess proteomic expression profiling as an advanced decision making tool or companion diagnostic are encouraged.

## Introduction

Psychiatric diagnoses are still based on criteria that focus on behavioral observation and symptom endorsement without corresponding biological validation. This contrasts with other fields of medicine, where diagnosis and treatment are often based not only on a sound clinical examination, but also biological tests based on validated biomarkers. Biological markers, or biomarkers, are quantitative measurements that provide information about biological processes, a disease state or about response to treatment (Food and Drug Administration (FDA)'s Biomarkers Research Group definition).^1^ There is increasing interest in developing feasibility studies for biomarker identification in mood disorders.^2^

Initial studies in schizophrenia, bipolar disorder and major depressive disorder have highlighted the potential utility of multiplex biomarker development. These studies were primarily non-hypothesis driven, based on an established immune mediator and cytokine quantification platforms, and were predominantly compared with a healthy control population. While there has been initial validation, replication and development of classification decision rules in a series of studies in schizophrenia,^3, 4, 5, 6^ the majority of studies have not been comparative within mood disorders and have not corrected for multiple testing and adjusted for covariates, thus limiting their replication potential and overall generalizability.^7, 8, 9, 10, 11, 12^ This study was conducted with Myriad RBM Human Multi-Analyte Profiling (MAP) platform to assess the feasibility of MAP in distinguishing (vs healthy controls) and differentiating subgroups of mood disorder patients.

## Materials and methods

This study was approved by the Mayo Clinic Institutional IRB (IRB number: 10-005352, principal investigator: Mark A. Frye). All participants provided written informed consent prior to enrollment, evaluation and biomarker blood draw.

### Subjects

**Figure 1:**
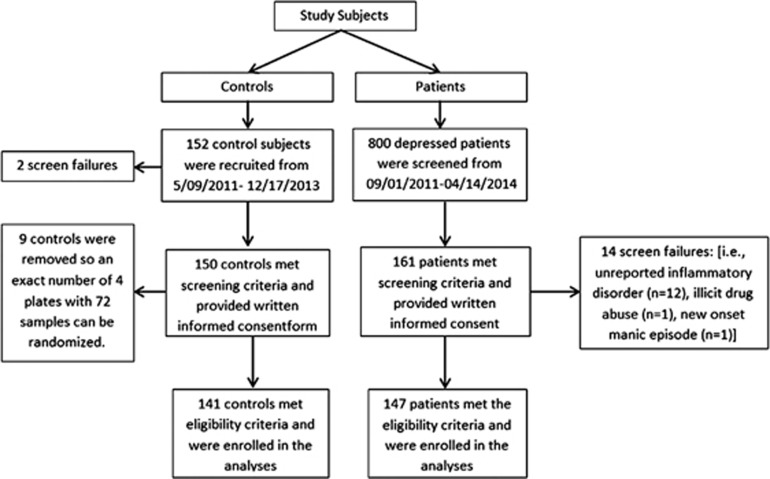
Subjects' enrollment flow diagram.

A consecutive sample of treatment-seeking adult (age 18–65) depressed patients from 9 May 2011 to 14 April 2014 were recruited from the Mayo Clinic Depression Center (Figure 1). Additional inclusion criteria included: diagnoses of major or bipolar I/II depression were confirmed by DSM IV TR Structured Clinical Diagnostic Interview (SCID).^13^ Exclusion criteria included: inability to provide written informed consent, other Axis I or II diagnoses that by clinical judgement were the main reason for seeking treatment, current substance use disorder determined by drug screen (except nicotine and caffeine), unipolar (UP) patients with first degree relative with bipolar disorders, acute unstable medical illness, inflammatory disease (that is, rheumatological, autoimmune), chronic pain, chronic use of non-steroidal or any anti-inflammatory drugs, systemic corticosteroids within the past 4 weeks, monoclonal antibody therapy within the past 3 months, acute infection or chronic infection requiring non-topical anti-infective agent, history of cancer with chemotherapy or radiation in the past year, and pregnant or lactating women.

Non-mood controls age 18–65 with no evidence of acute unstable medical illness, current or historic psychiatric diagnosis or first degree relative with psychiatric diagnosis were recruited from the community through newspaper, flyer, brochure and web-based advertisement (*n*=141).

### Protocol for drug screening

A urine drug screen was performed by study personnel using a One Step Multi-Drug Urine Test Panel kit (W.H.P.M., Irwindale, CA, USA) to screen for any current illicit substances after participants signed the study consent form. If illicit substances were detected in mood subjects, they were excluded from study unless the substance was medically prescribed (that is, benzodiazepines (Table 1) and stimulants (*n*=1)). If illicit substances were detected in healthy controls or patients, they were excluded from study (*n*=1). If a participant was currently an inpatient and completed a drug screen during their inpatient stay, the results of that urinalysis were used instead of requiring the participant to complete a second drug screen.

### Clinical assessment

Clinical assessments completed at the time of blood draw included: Inventory For Depressive Symptoms (IDS),^14^ the Young Mania Rating Scale (YMRS),^15^ Patient Health Questionnaire (PHQ-9),^16^ Generalized Anxiety Disorder 7-item scale (GAD-7)^17^ and Alcohol Use Disorders Identification Test (AUDIT).^18^ Current medications were recorded at the time of blood draw from the patient's electronic medical record and/or from the patient directly. In addition to current urine toxicology evaluation, additional substance use was quantified specifically lifetime alcohol use (self-report of never, occasional, regular and abuse), current cigarette smoking (yes/no) and lifetime history of illicit drug abuse (yes/no).

### Multi-analyte profiling

Myriad RBM is a CLIA-certified biomarker testing laboratory based in Austin, TX, USA. Their DiscoveryMAP is a quantitative multiplexed immunoassay service product, based on Luminex xMAP technology platform (for more information about the development of this platform see Supplementary online-text).^19, 20^ The current platform, based initially on pharmaceutical and biotechnological opportunities, focused on immune mediator and cytokine quantification for drug development has continued to expand and now includes 320 proteins (see Supplementary eTable 1).^21, 22, 23^ These same inflammatory and immune mediated biomarkers are increasingly recognized in the underlying neurobiology of mood disorders.^7, 8, 9, 10, 11, 12^

Using standard phlebotomy techniques and established laboratory protocol, the samples (~7.5 ml) were collected into serum-separating tubes. No adverse events were reported. Fasting vs non–fasting status and collection time (0600–1730 hours) was recorded on each sample. The time from blood draw to blood sample freeze was less than 1 h for all samples. All specimens collected at Mayo Clinic were packaged and sent overnight on dry ice to Myriad RBM. Shipped samples contained no direct identifiers and Myriad RBM was blind to plate identification and case vs control status.

About 288 serum samples (141 controls, 52 unipolar, 49 bipolar II, 46 bipolar I) were randomized to 4 plates (72 wells × 4). There was no difference in plate by group allocation and age of sample (from collection to analysis, R Kaldate, personal communication). Proteins (*n*=320) were measured in 250 μl serum samples using the DiscoveryMAP multiplexed immunoassays in the CLIA-certified laboratory at Myriad RBM. Assays were calibrated using duplicate eight-point standard curves and raw intensity measurements were converted to absolute protein concentrations using proprietary software. Assay performance was verified using quality control samples at low, medium and high levels for each analyte. All standard and quality control samples were analyzed in a complex matrix to match the sample background. All study protocols are in compliance with Standards for Reporting of Diagnostic Accuracy (STARD) initiative.^24^

### Statistical analysis

The relationships between clinical/demographic variables and diagnosis of mood disorder (cases vs controls) were modeled first using logistic regression models, testing the relationship of individual variables using likelihood ratio tests. Multinomial regression was used to model the relationship of the clinical/demographic variables with the mood disorder diagnoses (BP-I, BP-II and UP), and likelihood ratio tests were used to evaluate statistical significance.

For proteins with measurements below the Lower Limit of Quantification (LLOQ), to test for a relationship between protein levels and the clinical/demographic factors listed in (Supplementary eTable 2 in the Supplement), values less than the LLOQ were set to LLOQ/2. Analyte levels ⩾90% (*n*=48) below the LLOQ were excluded from analysis, leaving 272 proteins included in the study. There were also a few values above the upper limit of detection and they were set to the upper limit of detection. Since this imputation led to a distribution with a point mass at LLOQ/2 for some of the proteins, non-parametric tests were used to test for association between protein levels and covariates. Wilcoxon tests were used for categorical predictors, while Spearman correlation was used for continuous predictors, with an exact test approximation based on Monte Carlo simulation.

A number of the clinical/demographic variables were associated with levels of certain proteins (Supplementary eTable 2 in the Supplement). Therefore, when evaluating the association of diagnosis with protein levels, models were adjusted for current smoking status, history of illicit drug use, age, body mass index and years of education by including these variables as covariates. While not different in case vs control or within case analyses, we also adjusted for gender, lifetime alcohol use and fasting status because of their potential association with proteins. To model the relationships between diagnosis and proteins, first multinomial logistic regression was used to evaluate whether protein levels differ among the four diagnosis categories (BP-I vs BP-II vs UP vs control). Rather than imputing protein values below the LLOQ, the effect of the protein was modeled using two parameters corresponding to: (1) an indicator of a protein value being below the LLOQ, and (2) the actual protein value if the protein value was above the LLOQ, or zero otherwise (that is, the interaction of I*protein level, where I denotes the indicator variable for protein level>LLOQ). Sensitivity analyses were conducted imputing protein values less than the LLOQ using LLOQ/2, with similar results (data not shown). *P*-values were corrected for multiple testing using the Bonferroni method, thus *P*=0.05/272=1.84e−04 was considered statistically significant.

A series of *post hoc* pairwise comparisons between particular diagnoses (for example, BP-I+BP-II+UP vs controls, BP-I+BP-II vs controls, BP-I vs controls) were used to identify specific differences between diagnostic groups that contributed to significant results in the multinomial analyses of the four groups. For the pairwise comparisons, logistic regression was used to model probability of the two diagnoses using individual proteins as predictors, adjusting for the same covariates as in the multinomial analyses. To address multiple testing in these *post hoc* comparisons, the Bonferroni method was used, by further correcting the experiment-wise control of type I error corrected for 272 proteins (that is, 1.84e−04). Because only particular pairs of diagnoses were compared, three comparisons were accounted for in this multiple testing correction (*P*=1.84e−04/3=6.13e−05). While this was a pilot study, we have 80% power to detect odds ratios for predicting: mood disorders vs controls, bipolar disease vs controls and bipolar I disease vs controls of 1.72, 1.84 and 2.18 per s.d. increase in protein level assuming a type I error rate of 1.84E−04 (that is, the Bonferonni-corrected 5% error rate). While medication status was not considered in the primary analysis due to a high degree of confounding with diagnosis, a visual inspection of protein level differences was conducted by medication status (yes vs no for antipsychotics, lithium, sedatives/hypnotics, antidepressants and antiepileptic mood stabilizers) for each protein that was significantly different by diagnostic group.

To evaluate the predictive performance of the models, the area under the curve (AUC) of the receiver operating characteristic (ROC) curve was calculated from the logistic regression models based on the C-statistic. All analyses were completed using R v3.1.1 (Vienna, Austria); multinomial models were fit using 'nnet' R package v7.3-8 (New York, NY, USA), and C-statistic calculated using the 'rms' R package v4.2-1 (Nashville, TN, USA).

## Results

As shown in (Table 1), cases had significantly higher age, body mass index, current smoking and history of illicit drug use, as well as less years of education than controls. For all the clinical assessment comparisons, there were significant differences between cases and controls, but no difference between cases in moderate symptoms of depression, anxiety and alcohol screen; there was, however, a higher score on the YMRS, implying mix depressive symptoms in bipolar II patients.

**Figure 2:**
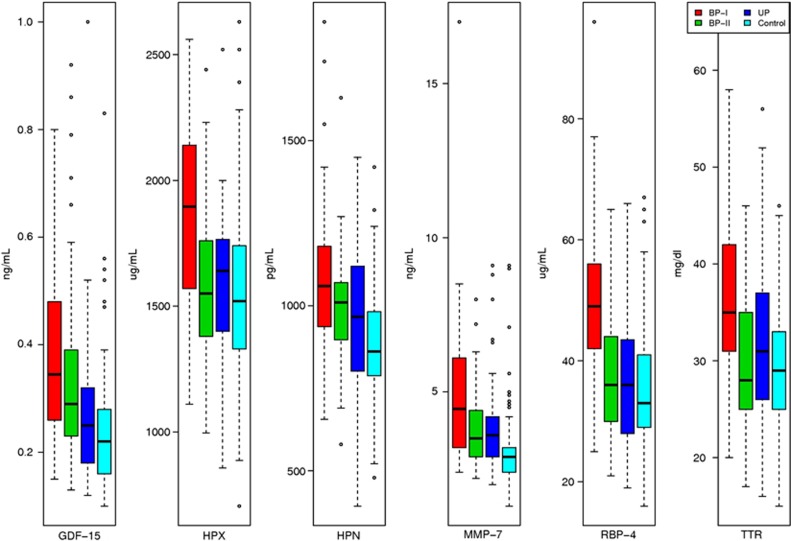
Comparison of proteins levels among groups (BP-I= 46, BP-II=49, UP=52, controls=141). All six proteins levels were higher in BP-I vs all other compared groups and controls. Five proteins were significantly different in BP-I vs controls GDF-15 *P*=0.0278, HPX *P*=0.0221, HPN *P*=0.0156, RBP-4 *P*=0.0001 and TTR *P*=0.0012. BP, bipolar depression; GDF-15, growth differentiation factor 15; HPN, hepsin; HPX, hemopexin; MMP-7, matrix metalloproteinase-7; RBP-4, retinol-binding protein 4; TTR, transthyretin.

Of the 272 proteins measured and analyzed from 288 samples (141 controls, 52 Unipolar, 49 Bipolar II, 46 Bipolar I), 73 showed nominally significant difference among the 4 compared groups at an uncorrected significance level (*P*<0.05. see bold font in Supplementary Table 1); 6 of those proteins showed statistically significant differences after Bonferroni correction (*P<*0.05/272=1.84e−04) (growth differentiation factor 15 (GDF-15), hemopexin (HPX), hepsin (HPN), matrix metalloproteinase-7 (MMP-7), retinol-binding protein 4 (RBP-4) and transthyretin (TTR)). Figure 2 shows the values of those 6 proteins by diagnosis, demonstrating that the protein levels were higher in BP-I vs all other compared groups and controls. As presented in Table 2, a series of three *post hoc* analyses were performed identifying greater statistical differences in the bipolar I vs control analyses. GDF-15, RBP-4 and TTR were good predictors of BP-I with ROC-AUC of 0.81, while HPX and HPN were fair predictors of BP-I with ROC-AUC of 0.74 and 0.78, respectively. The significant protein means and s.d. in each group, as well as fold changes between compared groups is demonstrated in Table 3.

For the six proteins significantly associated with mood disorders, values of proteins by diagnosis and medication groups (antipsychotics, lithium, antiepileptic mood stabilizers, antidepressants, sedatives/hypnotics) are presented (Supplementary eFigure 1 in the Supplement). These comparisons suggest that the effect may be driven by the diagnostic group, rather than medication. Although some differences appear to be further augmented by certain medications, differences between BP-I cases and controls (not on medication) appear to still be present for patients that are currently not taking these medications.

## Discussion

To our knowledge, this is the first study to assess the feasibility of high throughput multiplexed immunoassay technology (that is 272 proteins) for studying mood disorders. Taking advantage of an established platform based initially on immune mediator and cytokine quantification, increasingly recognized in the underlying neurobiology of mood disorders,^4, 5^ we have identified six proteins, all expressed in brain,^25, 26^ that distinguish mood disorder patients, particularly bipolar I patients from healthy controls. After adjusting for several possible confounders, 73 proteins differed among the 4 studied groups at an uncorrected significance level (see Supplementary Table 2), with GDF-15, HPX, HPN, MMP-7, RBP-4 and TTR showing statistically significant differences after conservative Bonferroni correction. Furthermore, GDF-15, RBP-4 and TTR were good predictors of BP-I with ROC-AUC>0.8. Although these discovery results need to be replicated in independent samples, this study demonstrates feasibility of a multiplex blood-based testing for bipolar disorder. This investigation has both identified new proteins possibly implicated in mood disorder and further refines our previous understanding of specific proteins in mood disorder.

GDF-15 plays a role as a growth factor as well as immune modulator, and has been implicated in cognitive decline.^27, 28^ HPX is a type II acute phase reactant glycoprotein and functions to bind heme, facilitate antioxidation and maintain iron homeostasis.^29^ HPX increases in preclinical early life stress models,^30^ and has been shown previously to be significantly elevated in bipolar and schizophrenic patients in comparison to controls.^31^ HPN is a type II membrane serine protease and might play a role in blood coagulation.^32^ To our knowledge, HPN association with mood disorders is reported here for the first time. MMP-7 is a metalloproteinase and its functions include breaking down of extracellular matrix, degrading proteoglycans, fibronectin, elastin and casein.^33^ MMP-7 cleaves synaptosomal-associated protein of 25 kDa (SNAP-25), an intraneuronal protein that is important for neurotransmitter release.^34^ This gene (rs6039769 variant) was also reported to be associated with early onset bipolar disorder.^35^

RBP-4 is a mainly expressed in liver with a primary function to transport retinol (vitamin A) from the liver to peripheral tissues.^36^ Vitamin A is essential for the brain to facilitate learning, memory and cognition^37^ and Rbp4^−^/^−^ knockout mice have cortical and hippocampal (CA3) neuronal loss and gliosis.^38^ Although a vitamin A bipolar disorder hypothesis has not been studied before, an earlier study reported low maternal vitamin A and an association with schizophrenia among exposed offsprings.^39^ There is one prior study that reported decreased RBP-4 is unipolar depressed patients; there was no bipolar comparison group.^40^

TTR is a homotetrameric protein synthesized by the liver, retinal pigment epithelium and choroid plexus.^41^ Present in cerebrospinal fluid (CSF), TTR transports tetraiodothyronine (T4) across blood–brain barrier.^42^ *TTR* locus, 18q12, has been implicated in bipolar disorder in a Danish pedigree of bipolar patients.^43^ Chronic administration of lithium to rats caused a 16-fold decrease in TTR,^44^ which differs from our data that suggest lithium-treated patients had an increase in TTR. Interestingly, neuropeptide Y has been found to be increased in both TTR knockout rats,^45^ and after prolonged lithium administration.^46^ Several studies have reported reduced CSF TTR in depressed patients,^47, 48, 49^ but this has not been confirmed in other investigations.^50^ In a recent clinical trial, it is been shown that bipolar depressed female patients treated with high dose levothyroxine (mean dose 325 μg) had a significant reduction in depressive symptoms in comparison to adjunctive placebo; our data could explain the therapeutic mechanism-of-action (that is, greater ability to transport more T4 via TTR to brain).^51^ However, TTR knockout mouse models are associated with reduced depressive-like behavior, which may be related to increased noradrenergic modulation in limbic forebrain.^52^ Interestingly, RBP-4 transports vitamin A by forming a complex with TTR (RBP-4/TTR). Taken together, RBP-4/TTR complex, thyroxine and vitamin A are all present in the CSF and participate in brain maturation and, cognitive, acquisition of memory and behavioral activities and may be implicated in mood disorders.^50^

Although this is the first attempt to study both bipolar I/II and unipolar depression, other studies have utilized MAP of specific mood disorders. Schwarz *et al.*,^4^ reported 20 proteins that were differentially expressed in pre-symptomatic BP subjects (*n*=110) vs controls. However, this study by Schwarz and colleagues did not correct for multiple tastings in the analysis and was not assessing symptomatic (i.e., depressed) patients. Domenici *et al.*,^3^ reported that insulin and MMP-9 were significantly higher in patients with major depressive disorder (*n*=245), vs controls. Although the *P*-value threshold was corrected for multiple testing, current mood state or symptom severity of the cases were not reported. Herberth *et al.*^53^ comparing euthymic BP patients (*n*=32) vs controls, identified three proteins (chemokine C-C motif ligand 2, endothelin-1, macrophage migration inhibitory factor) that showed statistically significant difference after correcting for multiple testing. While Stelzhammer *et al.*,^54^ performed a similar study on antidepressant drug-naïve unipolar patients (*n*=38) and identified 11 and 2 differentially expressed proteins utilizing multi-analyte profiling platform and liquid chromatography-mass spectrometry, respectively,^48^ the results were not corrected for multiple testing. The top results from both aforementioned studies did not match with our top findings.^53^ Haenisch *et al.*,^55^ reported 26 proteins, including MMP-7, which were differentially expressed in bipolar patients (*n*=17) vs controls. Similar to our data, the majority of these patients were BP-I with current or recent depression. Taken together, these previous studies either did not consider mood state in phenotyping, would likely not retain statistical significance after accounting for multiple testing correction or did not have access to this large of a proteomic platform.

The strengths of this study include a single recruitment site for depression treatment-seeking patients and rigorous exclusion criteria to eliminate non-specific inflammatory contributions from systemic illness and anti-inflammatory/biotic drug therapy. While there was no evidence of different level of symptom severity of anxiety, depression, alcohol use, between case groups, we did not conduct a protein level analysis with index episode duration, previous treatment trials nor SCID lifetime assessment of psychiatric diagnoses. The lifetime assessment of manic illness burden (that is, episodes), history of psychosis or age of illness onset may have provided greater understanding of the striking BP-I proteomic expression. The multiple correction testing was conservative. Furthermore, the more conservative adjustment for multiple covariates not commonly done in cross-sectional biomarker mood studies (fasting status, years education, lifetime drug abuse history and lifetime alcohol use) may have reduced power. Nonetheless, even after adjustment for these variables, this discovery has identified a number of proteins that differ significantly in patients with mood disorders, in particular bipolar I disorder.

One limitation of this study relates to the cross-sectional design without serial measurement. A second measurement when euthymic could address mood state-dependent proteomic expression, and clarify whether the proteomic expression is representative of bipolar depression, or trait or burden of previous mania/ hypomania-associated brain changes.^56, 57^ Another limitation of this study relates to the lack of statistical analyses, evaluating the effects of medication use on the observed protein level differences between diagnostic groups. This was not formally tested because of the lack of drug-naïve patients, and small sample size. Our exploratory descriptive data (Supplementary eFigure 1 in the supplements) suggest that medication status was not the primary driver of protein expression; however, this was not rigorously assessed. While disease, drug or an interaction between the two can change analyte or proteomic expression, none of the identified proteins have been identified, to our knowledge, in a drug mechanism-of-action or pharmacokinetic/pharmacodynamically mediated therapeutic drug response. The lack of association with thyroid hormone replacement, which was more prevalent in our BP-I vs BP-II and UP patient groups, and TTR has been confirmed.^58, 59^

While patients in the population are rarely treatment naïve, conducting similar studies in first index episode major depression where bipolar disorder is in the differential diagnosis has merit. Results of such studies could have significant clinical implications, aiding clinicians in selecting unimodal antidepressants vs FDA-approved bipolar depression treatments.^60^ Additional biomarker clinical verification (that is, replication of proteins distinguishing mood disorder subtypes) and utility studies (that is, proteomic-based clinical outcome or decision tool) are encouraged. A proteomic-based differential diagnosis as an advanced decision making tool or companion diagnostic to guide evidence-based algorithms for mood stabilizer vs unimodal antidepressant therapy would have great clinical impact.

In conclusion, the results of this feasibility study support the possibility of developing a diagnostic test using the discovered biomarkers, which need to be validated, to help facilitate accurate diagnosis and rapid treatment initiation with improved clinical outcomes. Further functional studies of the identified proteins will increase our understanding of the pathophysiology of mood disorders, which may lead to the discovery of novel pharmacological targets.

## Figures and Tables

**Table 1:** Demographics and clinical characteristics

		N *(%) or value*					P*-value*	
		*BP-I (*N=*46)*	*BP-II (*N=*49)*	*UP (*N=*52)*	*Controls (*N=*141)*	*Total (*N=*288)*	*Cases vs controls*	*Between cases*
*Gender*								
	Male *N* (%)	22 (47.8%)	25 (51%)	22 (42.3%)	59 (41.8%)	128 (44.4%)	0.38	0.67
	Female *N* (%)	24 (52.2%)	24 (49%)	30 (57.7%)	82 (58.2%)	160 (55.6%)		

*Age*								
	*n*	46	49	52	141	288	<0.001	0.29
	Mean (s.d.)	41.5 (12.3)	37.3 (14.7)	38.4 (13.6)	33.8 (11.6)	36.5 (12.9)		

*BMI*								
	*n*	46	49	52	141	288	<0.001	0.71
	Mean (s.d.)	30.3 (8.1)	30.2 (7.6)	29.3 (5.3)	25.8 (4.7)	27.9 (6.3)		

*Years of education*								
	*n*	46	49	52	141	288	<0.001	0.91
	Mean (s.d.)	15.1 (3.6)	15.3 (2.7)	15 (2.6)	16.7 (2.7)	15.9 (2.9)		

*Current smoking status*								
	No *N* (%)	35 (76.1%)	39 (79.6%)	36 (69.2%)	139 (98.6%)	249 (86.5%)	<0.001	0.48
	Yes *N* (%)	11 (23.9%)	10 (20.4%)	16 (30.8%)	2 (1.42%)	39 (13.5%)		

*Fasting*								
	No *N* (%)	39 (84.8%)	41 (83.7%)	48 (92.3%)	119 (84.4%)	247 (85.8%)	0.42	0.24
	Yes *N* (%)	7 (15.2%)	7 (14.3%)	3 (5.77%)	21 (14.9%)	38 (13.2%)		
	Missing	0 (0%)	1 (2.04%)	1 (1.92%)	1 (0.709%)	3 (1.04%)		

*Lifetime alcohol use*								
	No *N* (%)	10 (21.7%)	12 (24.5%)	10 (19.2%)	22 (15.6%)	54 (18.8%)	0.18	0.81
	Yes *N* (%)	36 (78.3%)	37 (75.5%)	42 (80.8%)	119 (84.4%)	234 (81.2%)		

*Lifetime illicit drug use*								
	No *N* (%)	21 (45.7%)	18 (36.7%)	22 (42.3%)	106 (75.2%)	167 (58%)	<0.001	0.67
	Yes *N* (%)	25 (54.3%)	31 (63.3%)	30 (57.7%)	35 (24.8%)	121 (42%)		

*Plate #*								
	1 *N* (%)	8 (17.4%)	19 (38.8%)	14 (26.9%)	31 (22%)	72 (25%)	0.37	0.24
	2 *N* (%)	12 (26.1%)	14 (28.6%)	13 (25%)	33 (23.4%)	72 (25%)		
	3 *N* (%)	13 (28.3%)	8 (16.3%)	15 (28.8%)	36 (25.5%)	72 (25%)		
	4 *N* (%)	13 (28.3%)	8 (16.3%)	10 (19.2%)	41 (29.1%)	72 (25%)		
		*BP-I (*N=*46)*	*BP-II (*N=*49)*	*UP (*N=*52)*	*Controls (*N=*141)*	*All mood (*N=*147)*	*Cases vs controls*	*Between cases*
*Clinical assessments*								
IDS-C	Mean (s.d.)	35.91 (11.4)	32.65 (11.16)	31.1 (11.73)	1.20 (1.76)	33.13 (11.5)	<0.001	0.115
PHQ-9	Mean (s.d.)	17.15 (7.17)	18.16 (6.39)	18.72 (5.34)	0.52 (.96)	18.04 (6.30)	<0.001	0.468
YMRS	Mean (s.d.)	2.95 (2.41)	3.53 (2.85)	2.09 (1.63)	0.27 (0.69)	2.84 (2.40)	<0.001	0.01
GAD-7	Mean (s.d.)	11.84 (6.34)	13.02 (5.39)	13.58 (4.85)	0.72 (1.43)	12.84 (5.55)	<0.001	0.296
AUDIT	Mean (s.d.)	4.67 (7.17)	4.98 (6.66)	5.05 (7.03)	2.73 (2.30)	4.91 (6.91)	<0.001	0.96

*Medications*								
Antipsychotics	*N* (%)	32 (70%)	23 (47%)	8 (15%)	0	63 (43%)	<0.001	<0.001
AED mood stabilizers	*N* (%)	29 (63%)	26 (53%)	3 (6%)	2	58 (39%)	<0.001	<0.001
Lithium	*N* (%)	25 (54%)	17 (35%)	0 (0%)	0	42 (29%)	<0.001	<0.001
Antidepressants	*N* (%)	21 (46%)	28 (57%)	46 (88%)	2	95 (65%)	<0.001	<0.001
Sedatives/hypnotics	*N* (%)	29 (63%)	22 (45%)	21 (40%)	0	72 (49%)	<0.001	0.062
Thyroxine supplement	*N* (%)	13 (28%)	7 (14%)	8 (15%)	4	28 (19%)	<0.001	0.125

Abbreviations: AED, AE mood stabilizers (antiepileptic mood stabilizers: valproate, lamotrigine, and carbamazepine); AUDIT, alcohol use disorders identification test; BMI, body mass index; BP, bipolar depression; GAD-7, generalized anxiety disorder 7-item scale; IDS-C, inventory for depressive symptoms-clinician rated; PHQ-9, patient health questionnaire; UP, unipolar; YMRS, young mania rating scale.

**Table 2:** Relationship between protein levels and mood disorder groups

*Protein*	*% Of below LLOQ*	*Protein vs diagnosis (all 4 groups)*	*All depressed subjects (BP-I, BP-II, UP) vs controls*			*BP depressed (BP-I+BP-II) vs controls*			*BP-I depressed vs controls*		
		P	P	*AUC*	*OR (95% CI)*	P	*AUC*	*OR (95% CI)*	P	*AUC*	*OR (95% CI)*
GDF-15	0	a0.022	1	b0.70	84.3 (0.357, 69 700)	a0.014	b0.76	764 (1.52,1 750 000)	a0.028	c0.81	3240 (1.3,40900000)
HPX	0	a0.041	1	0.62	1 (0.999,1)	1	0.63	1 (0.999,1)	a0.022	b0.74	1.0030 (1.0001,1.0066)
HPN	0	a0.039	1	0.69	1 (0.999,1.01)	a0.014	b0.73	1.0042 (1.0003,1.0089)	a0.016	b0.78	1.0053 (1.0003,1.0117)
MMP-7	0	a0.0036	a0.025	0.66	1.77 (1.02,3.46)	a0.0087	0.64	1.9 (1.05,3.97)	0.089	0.60	1.92 (0.981,4.59)
RBP-4	0	a0.0001	1	b0.74	1.03 (0.978,1.09)	0.522	b0.76	1.05 (0.992,1.12)	a0.0001	c0.81	1.11 (1.02,1.24)
TTR	0	a0.0048	1	0.63	1.05 (0.968,1.14)	1	0.68	1.07 (0.976,1.19)	a0.0012	c0.81	1.17 (1.02,1.39)

Abbreviations: AUC, area under the curve from the receiver operating characteristic curve; CI, confidence interval; LLOQ, lower limit of quantification; OR, odds ratio.

a Statistically significant (after Bonferroni correction).

b Fair prediction AUC= 0.7−0.79.

c Good prediction AUC⩾0.8.

**Table 3:** Significant protein mean levels and fold changes among compared groups

*Protein*	*Units*	*Control mean (s.d.)*	*All depressed mean (s.d.)*	*FC all depressed vs control*	*BP mean (s.d.)*	*FC BP vs control*	*BP-I mean (s.d.)*	*FC BP-I vs control*
GDF-15	ng ml^−1^	0.23 (0.1)	0.33 (0.16)	1.4	0.36 (0.16)	1.51	0.37 (0.14)	1.57
HPX	μg ml^−1^	1550.05 (320.65)	1683.38 (342.94)	1.09	1721.75 (368.09)	1.11	1854.57 (367.98)	1.2
HPN	pg ml^−1^	888.55 (164.28)	1014.19 (218.82)	1.14	1041.47 (212.83)	1.17	1099.65 (238.25)	1.24
MMP-7	ng ml^−1^	3.07 (1.12)	4.22 (1.9)	1.37	4.4 (2.05)	1.43	4.99 (2.51)	1.62
RBP-4	μg ml^−1^	35.49 (10.28)	41.49 (13.51)	1.17	43.64 (13.68)	1.23	49.96 (13.7)	1.41
TTR	mg dl^−1^	29.6 (6.36)	32.67 (8.46)	1.1	33 (8.28)	1.12	36.35 (8.38)	1.23

Abbreviations: BP, bipolar depression; FC, fold change; GDF-15, growth differentiation factor 15; HPN, hepsin; HPX, hemopexin; MMP-7, matrix metalloproteinase-7; RBP-4, retinol-binding protein 4; TTR, transthyretin.

FC is calculated based on the mean concentration of each protein in the cases group divided by that in the control group.
