# Supplementary material for: Feasibility of investigating differential proteomic expression in depression: implications for biomarker development in mood disorders
Source: Transl Psychiatry. 2015 Dec 8;5(12):e689–. doi: 10.1038/tp.2015.185 (PMC5068585; doi:10.1038/tp.2015.185)
Supplement: Supplementary Information [file tp2015185x1.doc]

**Supplements**

Summary:

This file consists of further information about the development of DiscoveryMAP® (online text), as well as a list of the measured proteins [highlighting the proteins that differed among the 4 compared groups at an uncorrected significance level (p<0.05)] (eTable 1.S), a table showing clinical/demographic variables that are associated with levels of the top associated proteins (eTable 2.S), and a set of figures showing protein level differences by medication status (eFigure 1.S).

Online-Text.

**Development of DiscoveryMAP®**

From a small sample volume, the DiscoveryMAP® platform provides reproducible, quantitative, multiplexed immunoassay data for hundreds of proteins. The units of the measured proteins are the standard units found in the literature, with ranges based on ~100 individuals with no apparent illness (with equal mixture of male and female, and average age of 35 with the minimum 20 and maximum 66). Reference standard is based upon clinically acceptable range and literature ranges when available. Reproducibility of the test is based on three levels of controls run in duplicate performed when each batch of samples is tested (a batch of samples is no larger than 72 samples). Each assay in the DiscoveryMAP® was developed as single test using a single Luminex microsphere set, to establish dynamic range and sensitivity necessary for each analyte. Finally, luminex multiplex technology (used in DiscoveryMAP®) has been already validated utilizing ELISA (producing results comparable to ELISA)[^1^](#_ENREF_1).

More information about the measured proteins by DiscoveryMAP® is available on Myriad website: <http://rbm.myriad.com/products-services/humanmap-services/human-discoverymap/>

eTable 1.S. List of measured proteins

**Bold:** 73 proteins differed among the 4 groups at an uncorrected significance level (p<0.05)

| **#** | **Unit** | **Protein** |
| --- | --- | --- |
| 1 | pg/mL | **6Ckine/** **chemokine (C-C motif) ligand 21** |
| 2 | ug/mL | Adiponectin |
| 3 | ng/mL | Adrenomedullin |
| 4 | pg/mL | Agouti-Related Protein |
| 5 | ng/mL | **Aldose Reductase** |
| 6 | ug/mL | **Alpha-1-acid glycoprotein 1** |
| 7 | ug/mL | Alpha-1-Antichymotrypsin |
| 8 | mg/mL | **Alpha-1-Antitrypsin** |
| 9 | ug/mL | **Alpha-1-Microglobulin** |
| 10 | mg/mL | Alpha-2-Macroglobulin |
| 11 | ng/mL | Alpha-Fetoprotein |
| 12 | pg/mL | Amphiregulin |
| 13 | ng/mL | Angiogenin |
| 14 | ng/mL | Angiopoietin-1 |
| 15 | ng/mL | Angiopoietin-2 |
| 16 | ng/mL | Angiopoietin-related protein 3 |
| 17 | ng/mL | Angiotensin-Converting Enzyme |
| 18 | ng/mL | Antileukoproteinase |
| 19 | ug/mL | **Antithrombin-III** |
| 20 | ug/mL | Apolipoprotein(a) |
| 21 | mg/mL | Apolipoprotein A-I |
| 22 | ng/mL | **Apolipoprotein A-II** |
| 23 | ug/mL | Apolipoprotein A-IV |
| 24 | ug/mL | **Apolipoprotein B** |
| 25 | ng/mL | **Apolipoprotein C-I** |
| 26 | ug/mL | Apolipoprotein C-III |
| 27 | ug/mL | **Apolipoprotein D** |
| 28 | ug/mL | Apolipoprotein E |
| 29 | ug/mL | Apolipoprotein H |
| 30 | ng/mL | AXL Receptor Tyrosine Kinase |
| 31 | pg/mL | B cell-activating factor |
| 32 | pg/mL | B Lymphocyte Chemoattractant |
| 33 | ng/mL | Beta Amyloid 1-40 |
| 34 | ng/mL | Beta Amyloid 1-42 |
| 35 | ug/mL | Beta-2-Microglobulin |
| 36 | ng/mL | Beta-microseminoprotein |
| 37 | pg/mL | Betacellulin |
| 38 | ng/mL | Brain-Derived Neurotrophic Factor |
| 39 | ng/mL | **C-Peptide** |
| 40 | ug/mL | C-Reactive Protein |
| 41 | ng/mL | Cadherin-1 |
| 42 | ng/mL | Cadherin-13 |
| 43 | ng/mL | Calbindin |
| 44 | U/mL | Cancer Antigen 125 |
| 45 | U/mL | Cancer Antigen 15-3 |
| 46 | U/mL | Cancer Antigen 19-9 |
| 47 | ng/mL | **Carbonic anhydrase 9** |
| 48 | ng/mL | Carcinoembryonic Antigen |
| 49 | ng/mL | **Carcinoembryonic antigen-related cell adhesion molecule 1** |
| 50 | ng/mL | Carcinoembryonic antigen-related cell adhesion molecule 6 |
| 51 | ng/mL | **Cartilage Oligomeric Matrix Protein** |
| 52 | ng/mL | Cathepsin B (pro) |
| 53 | ng/mL | Cathepsin D |
| 54 | ng/mL | CD5 Antigen-like |
| 55 | U/mL | CD27 antigen |
| 56 | ng/mL | CD 40 antigen |
| 57 | ng/mL | **CD40 Ligand** |
| 58 | ng/mL | CD163 |
| 59 | ug/mL | Cellular Fibronectin |
| 60 | ug/mL | **Ceruloplasmin** |
| 61 | ng/mL | **Chemerin** |
| 62 | ng/mL | Chemokine CC-4 |
| 63 | ng/mL | **Chromogranin-A** |
| 64 | pg/mL | Ciliary Neurotrophic Factor |
| 65 | ug/mL | **Clusterin** |
| 66 | ng/mL | Collagen IV |
| 67 | mg/mL | **Complement C3** |
| 68 | ug/mL | **Complement component C1q receptor** |
| 69 | ug/mL | **Complement Factor H** |
| 70 | ug/mL | Complement Factor H – Related Protein 1 |
| 71 | ng/mL | Cortisol |
| 72 | ng/mL | Creatine Kinase-MB |
| 73 | ng/mL | Cystatin-A |
| 74 | ng/mL | **Cystatin-B** |
| 75 | ng/mL | Cystatin-C |
| 76 | ng/mL | **Decorin** |
| 77 | ng/mL | Dickkopf-related protein 1 |
| 78 | ng/mL | Dipeptidyl peptidase IV |
| 79 | ng/mL | Dopamine beta-hydroxylase |
| 80 | ng/mL | **E-Selectin** |
| 81 | ng/mL | EN-RAGE/ S100 calcium binding protein A12 |
| 82 | ng/mL | Endoglin |
| 83 | ng/mL | Endostatin |
| 84 | pg/mL | Eotaxin-1 |
| 85 | pg/mL | Eotaxin-2 |
| 86 | pg/mL | Eotaxin-3 |
| 87 | pg/mL | Epidermal Growth Factor |
| 88 | ng/mL | **Epidermal Growth Factor Receptor** |
| 89 | pg/mL | Epiregulin |
| 90 | pg/mL | Epithelial cell adhesion molecule |
| 91 | ng/mL | **Epithelial-Derived Neutrophil-Activating Protein 78** |
| 92 | ng/mL | Factor VII |
| 93 | pg/mL | **Fas Ligand** |
| 94 | ng/mL | FASLG Receptor |
| 95 | ng/mL | Fatty Acid-Binding Protein, adipocyte |
| 96 | ng/mL | Fatty Acid-Binding Protein, heart |
| 97 | ng/mL | Fatty Acid-Binding Protein, liver |
| 98 | ng/mL | Ferritin |
| 99 | ug/mL | Fetuin-A |
| 100 | mg/mL | Fibrinogen |
| 101 | pg/mL | Fibroblast Growth Factor 4 |
| 102 | ng/mL | Fibroblast Growth Factor 21 |
| 103 | ng/mL | Fibroblast growth factor 23 |
| 104 | pg/mL | Fibroblast Growth Factor basic |
| 105 | ug/mL | Fibulin-1C |
| 106 | ug/mL | **Ficolin-3** |
| 107 | mIU/mL | Follicle-Stimulating Hormone |
| 108 | ng/mL | Galectin-3 |
| 109 | ng/mL | Gastric inhibitory polypeptide |
| 110 | ug/mL | Gelsolin |
| 111 | pg/mL | Glucagon |
| 112 | pg/mL | Glucagon-like Peptide 1, active |
| 113 | pg/mL | Glucagon-like Peptide 1, total |
| 114 | ng/mL | **Glucose-6-phosphate Isomerase** |
| 115 | ng/mL | **Glutathione S-Transferase alpha** |
| 116 | ng/mL | Glutathione S-Transferase Mu 1 |
| 117 | ng/mL | Glycogen phosphorylase isoenzyme BB |
| 118 | pg/mL | Granulocyte Colony-Stimulating Factor |
| 119 | pg/mL | Granulocyte-Macrophage Colony-Stimulating Factor |
| 120 | ng/mL | **Growth/differentiation factor 15** |
| 121 | ng/mL | Growth Hormone |
| 122 | pg/mL | Growth-Regulated alpha protein |
| 123 | mg/mL | Haptoglobin |
| 124 | pM | **HE4/** **WAP four-disulfide core domain 2** |
| 125 | ng/mL | Heat Shock Protein 60 |
| 126 | ng/mL | Heat-Shock protein 70 |
| 127 | ug/mL | **Hemopexin** |
| 128 | pg/mL | Heparin-Binding EGF-Like Growth Factor |
| 129 | ng/mL | Hepatocyte Growth Factor |
| 130 | ng/mL | Hepatocyte Growth Factor receptor |
| 131 | pg/mL | **Hepsin** |
| 132 | mIU/mL | Human Chorionic Gonadotropin beta |
| 133 | ng/mL | Human Epidermal Growth Factor Receptor 2 |
| 134 | mg/mL | Immunoglobulin A |
| 135 | U/mL | Immunoglobulin E |
| 136 | mg/mL | **Immunoglobulin M** |
| 137 | uIU/mL | **Insulin** |
| 138 | ng/mL | **Insulin-like Growth Factor-Binding Protein 1** |
| 139 | ng/mL | Insulin-like Growth Factor-Binding Protein 2 |
| 140 | ng/mL | Insulin-like Growth Factor-Binding Protein 3 |
| 141 | ng/mL | Insulin-like Growth Factor Binding Protein 4 |
| 142 | ng/mL | Insulin-like Growth Factor Binding Protein 5 |
| 143 | ng/mL | Insulin-like Growth Factor Binding Protein 6 |
| 144 | ng/mL | Insulin-like Growth Factor-Binding Protein 7 |
| 145 | ng/mL | Intercellular Adhesion Molecule 1 |
| 146 | ng/mL | Interferon alpha |
| 147 | pg/mL | Interferon gamma |
| 148 | pg/mL | Interferon gamma Induced Protein 10 |
| 149 | pg/mL | Interferon-inducible T-cell alpha chemoattractant |
| 150 | ng/mL | Interleukin-1 alpha |
| 151 | pg/mL | Interleukin-1 beta |
| 152 | pg/mL | Interleukin-1 receptor antagonist |
| 153 | pg/mL | Interleukin-1 receptor type 1 |
| 154 | pg/mL | Interleukin-2 |
| 155 | ng/mL | **Interleukin-1 receptor type 2** |
| 156 | pg/mL | Interleukin-2 receptor alpha |
| 157 | ng/mL | Interleukin-3 |
| 158 | pg/mL | Interleukin-4 |
| 159 | pg/mL | Interleukin-5 |
| 160 | pg/mL | **Interleukin-6** |
| 161 | ng/mL | Interleukin-6 receptor |
| 162 | ng/mL | Interleukin-6 receptor subunit beta |
| 163 | pg/mL | Interleukin-7 |
| 164 | pg/mL | Interleukin-8 |
| 165 | pg/mL | **Interleukin-10** |
| 166 | ng/mL | Interleukin-12 Subunit p40 |
| 167 | pg/mL | Interleukin-12 Subunit p70 |
| 168 | pg/mL | Interleukin-13 |
| 169 | ng/mL | Interleukin-15 |
| 170 | pg/mL | Interleukin-16 |
| 171 | pg/mL | Interleukin-17 |
| 172 | pg/mL | Interleukin-18 |
| 173 | ng/mL | Interleukin-18-binding protein |
| 174 | ng/mL | Interleukin-22 |
| 175 | ng/mL | Interleukin-23 |
| 176 | ng/mL | Interleukin-31 |
| 177 | ng/mL | Kallikrein 5 |
| 178 | pg/mL | Kallikrein-7 |
| 179 | ng/mL | Kidney Injury Molecule-1 |
| 180 | ng/mL | Lactoferrin |
| 181 | ng/mL | **Lactoylglutathione lyase** |
| 182 | ng/mL | **Latency-Associated Peptide of Transforming Growth Factor beta 1** |
| 183 | ng/mL | Lectin-Like Oxidized LDL Receptor 1 |
| 184 | ng/mL | Leptin |
| 185 | ng/mL | Leptin Receptor |
| 186 | ug/mL | Leucine-rich alpha-2-glycoprotein |
| 187 | ug/mL | Lumican |
| 188 | mIU/mL | Luteinizing Hormone |
| 189 | ng/mL | Macrophage Colony-Stimulating Factor 1 |
| 190 | pg/mL | **Macrophage-Derived Chemokine** |
| 191 | pg/mL | Macrophage Inflammatory Protein-1 alpha |
| 192 | pg/mL | Macrophage Inflammatory Protein-1 beta |
| 193 | pg/mL | **Macrophage Inflammatory Protein-3 alpha** |
| 194 | pg/mL | Macrophage inflammatory protein 3 beta |
| 195 | ng/mL | Macrophage Migration Inhibitory Factor |
| 196 | ng/mL | Macrophage-Stimulating Protein |
| 197 | pg/mL | Maspin |
| 198 | ng/mL | Mast/stem cell growth factor receptor |
| 199 | ng/mL | Matrix Metalloproteinase-1 |
| 200 | ng/mL | **Matrix Metalloproteinase-2** |
| 201 | ng/mL | Matrix Metalloproteinase-3 |
| 202 | ng/mL | **Matrix Metalloproteinase-7** |
| 203 | ng/mL | Matrix Metalloproteinase-9 |
| 204 | ng/mL | **Matrix Metalloproteinase-9, total** |
| 205 | ng/mL | Matrix Metalloproteinase-10 |
| 206 | nM | Mesothelin |
| 207 | pg/mL | MHC class I chain-related protein A |
| 208 | pg/mL | Monocyte Chemotactic Protein 1 |
| 209 | pg/mL | Monocyte Chemotactic Protein 2 |
| 210 | pg/mL | Monocyte Chemotactic Protein 3 |
| 211 | pg/mL | Monocyte Chemotactic Protein 4 |
| 212 | pg/mL | Monokine Induced by Gamma Interferon |
| 213 | ng/mL | Myeloid Progenitor Inhibitory Factor 1 |
| 214 | ng/mL | Myeloperoxidase |
| 215 | ng/mL | **Myoglobin** |
| 216 | pg/mL | **N-terminal prohormone of brain natriuretic peptide** |
| 217 | ng/mL | Nerve Growth Factor beta |
| 218 | pg/mL | Neurofilament heavy polypeptide |
| 219 | ng/mL | Neuron-Specific Enolase |
| 220 | ng/mL | **Neuronal Cell Adhesion Molecule** |
| 221 | ng/mL | Neuropilin-1 |
| 222 | ng/mL | **Neutrophil Activating Peptide 2** |
| 223 | ng/mL | Neutrophil Gelatinase-Associated Lipocalin |
| 224 | ng/mL | **Omentin** |
| 225 | ng/mL | Osteocalcin |
| 226 | ng/mL | Osteopontin |
| 227 | pM | Osteoprotegerin |
| 228 | ng/mL | P-Selectin |
| 229 | pg/mL | Pancreatic Polypeptide |
| 230 | ng/mL | **Pancreatic secretory trypsin inhibitor** |
| 231 | ng/mL | Paraoxonase-1 |
| 232 | ng/mL | Pentraxin-3 |
| 233 | ng/mL | **Pepsinogen I** |
| 234 | ug/mL | Peptidase D |
| 235 | pg/mL | Peptide YY |
| 236 | ng/mL | Periostin |
| 237 | ng/mL | Phosphoserine Aminotransferase |
| 238 | ng/mL | **Pigment Epithelium Derived Factor** |
| 239 | pg/mL | Placenta Growth Factor |
| 240 | ng/mL | Plasminogen Activator Inhibitor 1 |
| 241 | ng/mL | Platelet endothelial cell adhesion molecule |
| 242 | pg/mL | Platelet-Derived Growth Factor BB |
| 243 | ng/mL | **Progesterone** |
| 244 | ng/mL | Progranulin |
| 245 | pM | Proinsulin, Intact |
| 246 | pM | Proinsulin, Total |
| 247 | ng/mL | Prolactin |
| 248 | ng/mL | **Prostasin** |
| 249 | ng/mL | Prostate-Specific Antigen, Free |
| 250 | ng/mL | Prostate Specific Antigen, total |
| 251 | ng/mL | Protein DJ-1 |
| 252 | ng/mL | Protein S100-A4 |
| 253 | ng/mL | **Pulmonary and Activation-Regulated Chemokine** |
| 254 | ng/mL | Pulmonary surfactant-associated protein D |
| 255 | ng/mL | Receptor for advanced glycosylation end products |
| 256 | ng/mL | Receptor tyrosine-protein kinase erbB-3 |
| 257 | ng/mL | Resistin |
| 258 | ug/mL | **Retinol-binding protein 4** |
| 259 | ng/mL | S100 calcium-binding protein B |
| 260 | ng/mL | Selenoprotein P |
| 261 | mg/dl | **Serotransferrin** |
| 262 | ng/mL | Serum Amyloid A Protein |
| 263 | ug/mL | **Serum Amyloid P-Component** |
| 264 | nmol/L | **Sex Hormone-Binding Globulin** |
| 265 | ng/mL | Sortilin |
| 266 | ng/mL | **Squamous Cell Carcinoma Antigen-1** |
| 267 | ng/mL | ST2/ Interleukin-1 receptor-like 1 |
| 268 | pg/mL | Stem Cell Factor |
| 269 | pg/mL | Stromal cell-derived factor-1 |
| 270 | ng/mL | Superoxide Dismutase 1, soluble |
| 271 | ng/mL | T-Cell-Specific Protein RANTES |
| 272 | pg/mL | T Lymphocyte-Secreted Protein I-309 |
| 273 | ug/mL | Tamm-Horsfall Urinary Glycoprotein |
| 274 | ng/mL | Tenascin-C |
| 275 | ng/mL | Tenascin-X |
| 276 | ng/mL | Testosterone, Total |
| 277 | ug/mL | Tetranectin |
| 278 | ug/mL | **Thrombin-Activatable Fibrinolysis** |
| 279 | ng/mL | Thrombomodulin |
| 280 | ng/mL | **Thrombospondin-1** |
| 281 | ug/mL | Thrombospondin-4 |
| 282 | ng/mL | Thymus and activation-regulated chemokine |
| 283 | pg/mL | Thymus-Expressed Chemokine |
| 284 | ng/mL | Thyroglobulin |
| 285 | uIU/mL | **Thyroid-Stimulating Hormone** |
| 286 | ug/mL | **Thyroxine-Binding Globulin** |
| 287 | ng/mL | **Tissue Inhibitor of Metalloproteinases 1** |
| 288 | ng/mL | **Tissue Inhibitor of Metalloproteinases 2** |
| 289 | ng/mL | Tissue Inhibitor of Metalloproteinases 3 |
| 290 | ng/mL | Tissue type Plasminogen activator |
| 291 | ng/mL | TNF-Related Apoptosis-Inducing Ligand Receptor 3 |
| 292 | ug/mL | Transferrin receptor protein 1 |
| 293 | pg/mL | **Transforming Growth Factor alpha** |
| 294 | pg/mL | Transforming Growth Factor beta-3 |
| 295 | mg/dl | **Transthyretin** |
| 296 | ug/mL | **Trefoil Factor 3** |
| 297 | pg/mL | Tumor Necrosis Factor alpha |
| 298 | pg/mL | Tumor Necrosis Factor beta |
| 299 | ng/mL | Tumor necrosis factor ligand superfamily member 12 |
| 300 | ng/mL | Tumor necrosis factor ligand superfamily member 13 |
| 301 | pg/mL | **Tumor Necrosis Factor Receptor I** |
| 302 | ng/mL | Tumor necrosis factor receptor 2 |
| 303 | ng/mL | Tyrosine kinase with Ig and EGF homology domains 2 |
| 304 | pg/mL | Urokinase-type Plasminogen Activator |
| 305 | ng/mL | **Urokinase-type plasminogen activator receptor** |
| 306 | ng/mL | Vascular Cell Adhesion Molecule-1 |
| 307 | pg/mL | Vascular Endothelial Growth Factor |
| 308 | ng/mL | Vascular endothelial growth factor B |
| 309 | ng/mL | Vascular Endothelial Growth Factor C |
| 310 | pg/mL | Vascular endothelial growth factor D |
| 311 | pg/mL | Vascular Endothelial Growth Factor Receptor 1 |
| 312 | ng/mL | **Vascular Endothelial Growth Factor Receptor 2** |
| 313 | ng/mL | **Vascular endothelial growth factor receptor 3** |
| 314 | pg/mL | Visceral adipose tissue – derived serpin A12 |
| 315 | ng/mL | Visfatin |
| 316 | ug/mL | Vitamin D-Binding Protein |
| 317 | ug/mL | **Vitamin K-Dependent Protein S** |
| 318 | ug/mL | **Vitronectin** |
| 319 | ug/mL | von Willebrand Factor |
| 320 | ng/mL | YKL-40/ Chitinase-3-like protein 1 |

eTable 2.S. Clinical/demographic variables associated with levels of certain proteins.

Y: yes, N: no, BMI: Body Mass Index

|  | p-value of association | | | | | | | | |
| --- | --- | --- | --- | --- | --- | --- | --- | --- | --- |
| **protein** | **Gender** | **Lifetime Alcohol use (Y/N)** | **Current Smoking (Y/N)** | **Prior Suicide attempt (Y/N)** | **Fasted (Y/N)** | **Lifetime Illicit drug use (Y/N)** | **Age** | **BMI** | **Years of education** |
| (GDF-15) | 7.16E-02 | 5.80E-01 | 2.12E-02 | 6.80E-03 | 2.01E-02 | 1.60E-04 | 4.62E-22 | 9.68E-06 | 9.25E-02 |
| (HPX) | 1.33E-03 | 3.82E-01 | 5.12E-01 | 5.50E-03 | 4.24E-01 | 2.43E-01 | 5.21E-03 | 2.97E-08 | 1.13E-01 |
| (HPN) | 5.67E-01 | 6.24E-01 | 3.90E-03 | 1.95E-03 | 9.49E-01 | 9.88E-03 | 5.95E-04 | 2.88E-04 | 8.13E-03 |
| (MMP-7) | 1.61E-02 | 6.17E-01 | 7.60E-04 | 1.20E-03 | 3.12E-01 | 1.96E-01 | 6.16E-08 | 4.81E-04 | 4.44E-03 |
| (RBP-4) | 2.92E-03 | 3.45E-01 | 2.14E-01 | 5.09E-03 | 9.57E-01 | 8.20E-03 | 6.66E-03 | 1.49E-04 | 2.59E-01 |
| (TTR) | 1.10E-04 | 7.54E-01 | 1.87E-01 | 2.30E-02 | 9.99E-01 | 4.49E-02 | 8.83E-01 | 1.06E-01 | 2.05E-01 |

eFigure 1.S (A-E) Protein levels by medication status when comparing BP-I with medication, BP-I without medication, and Controls

AE= Antiepileptic mood stabilizers

eFigure 1.S-A: Antidepressant


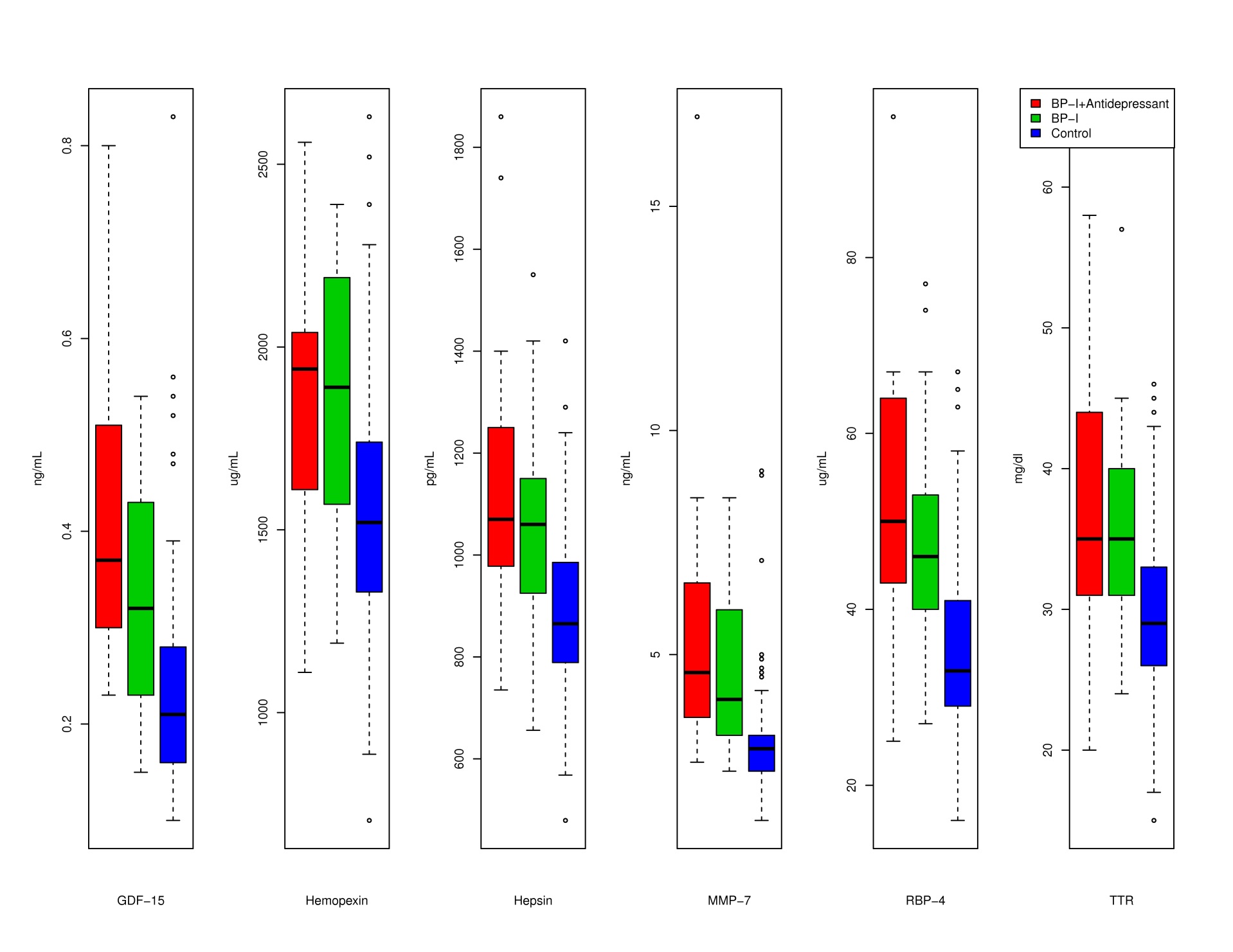


eFigure 1.S-B: Antiepileptic


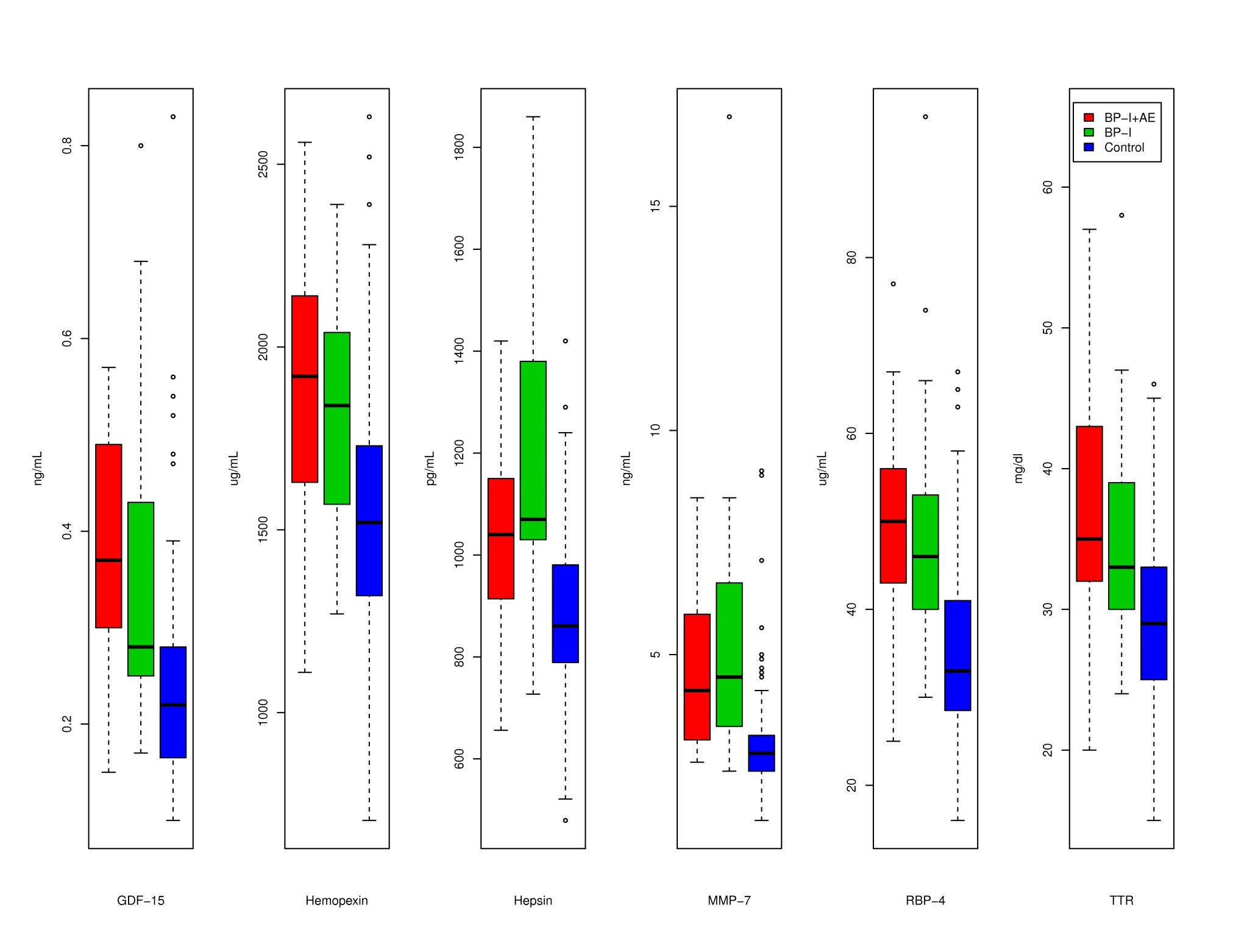


eFigure 1.S-C: Antipsychotic


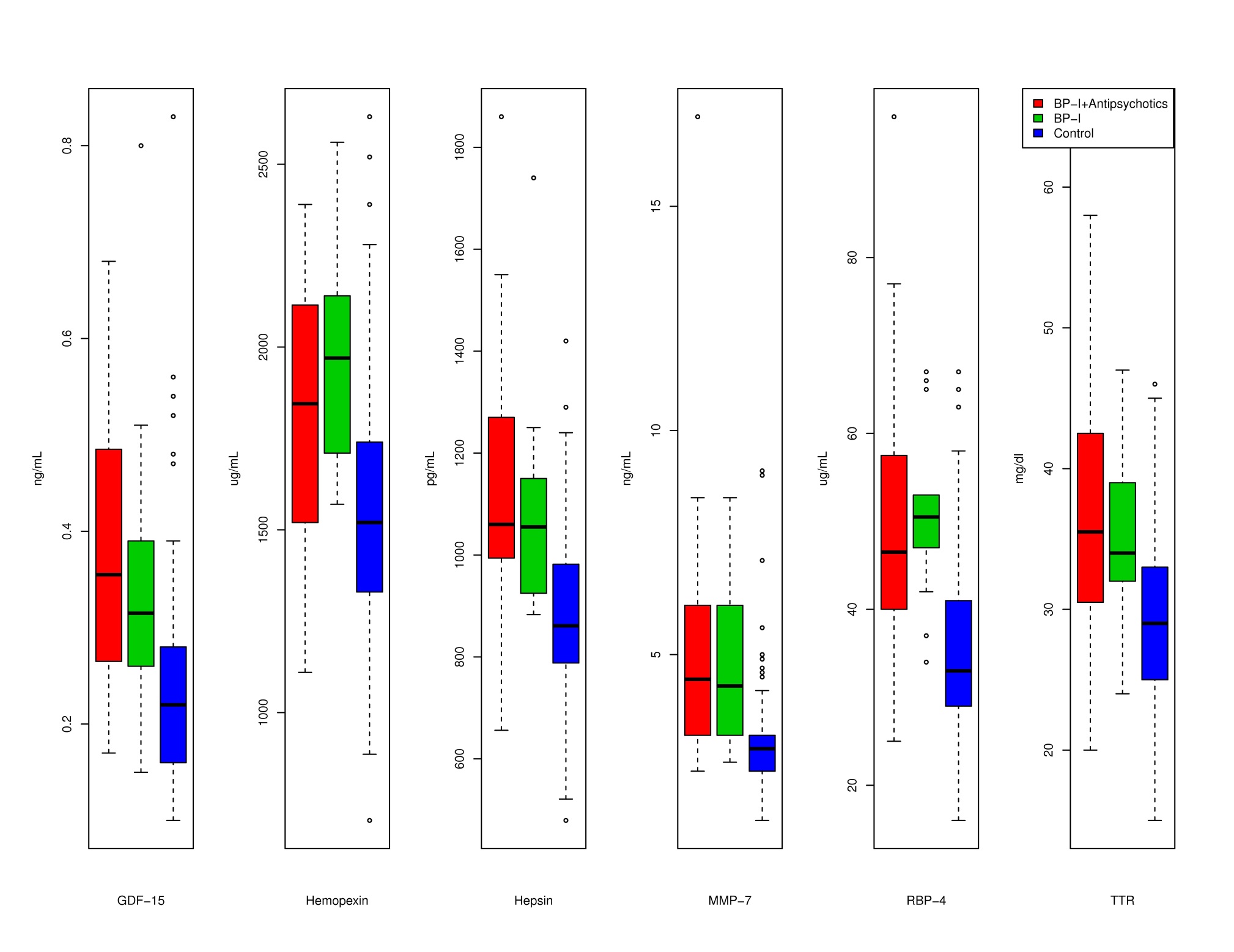


eFigure 1.S-D: Lithium

**
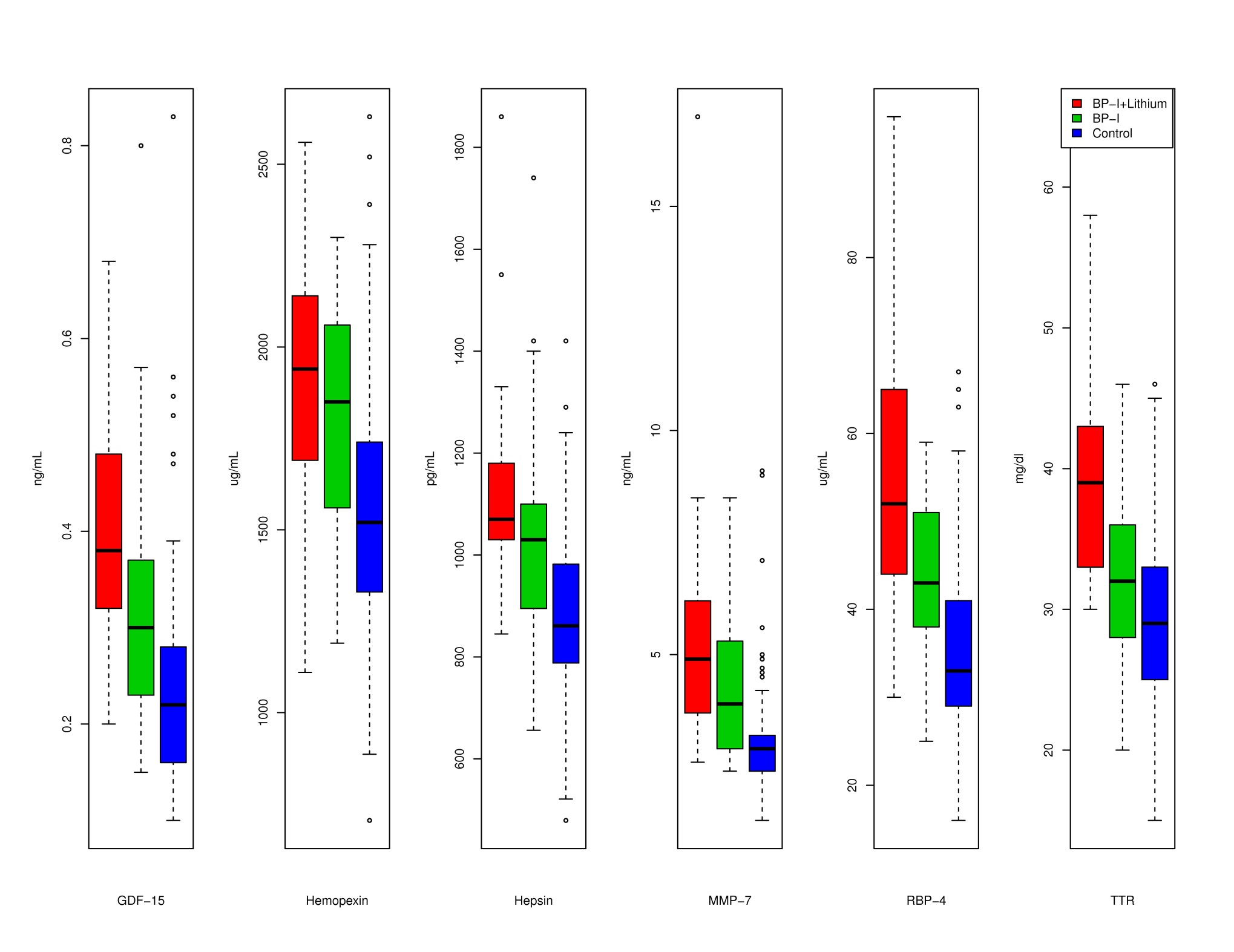
**

eFigure 1.S-E: Sedatives

**
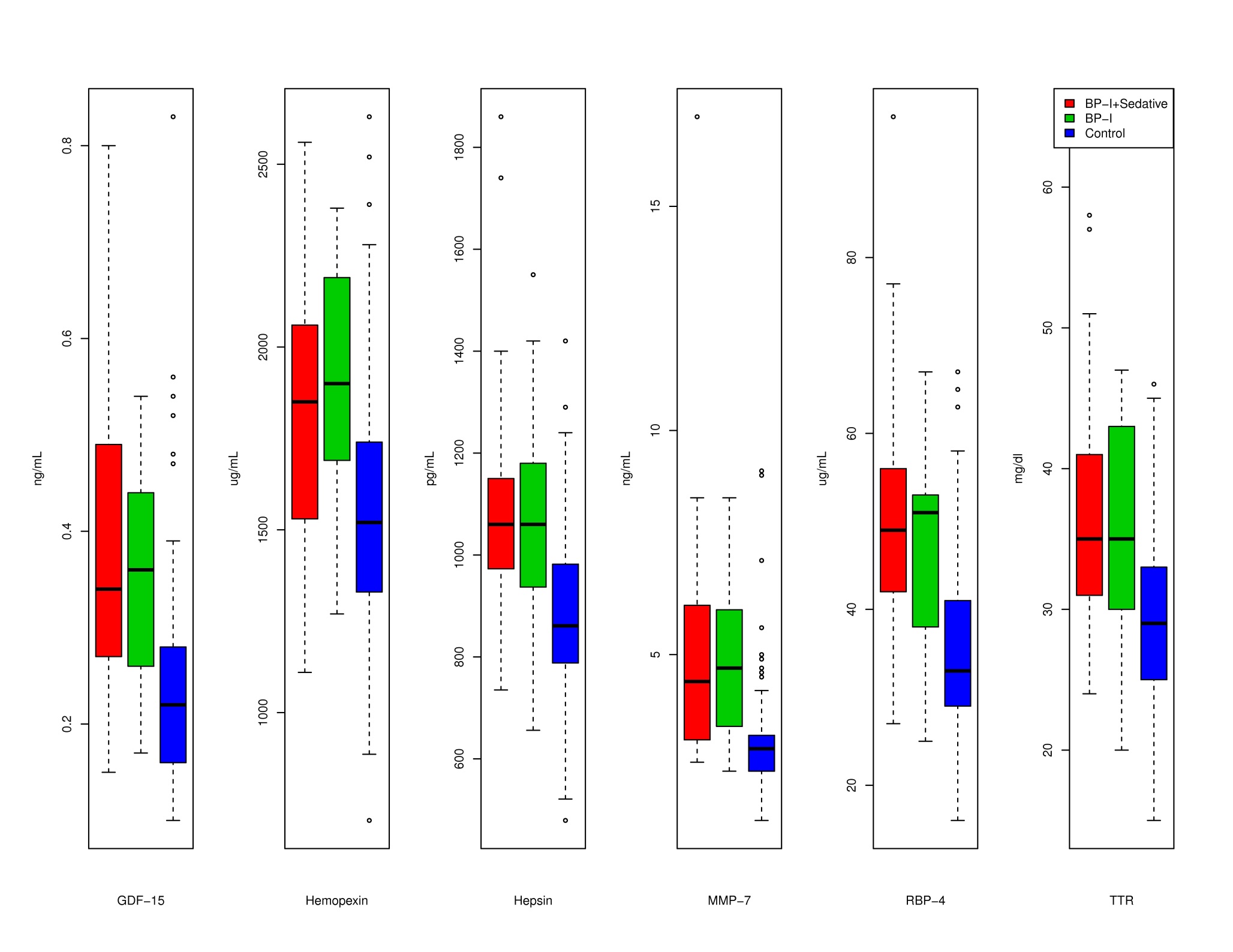
**

**References:**

1. Dupont NC, Wang K, Wadhwa PD, Culhane JF, Nelson EL. Validation and comparison of luminex multiplex cytokine analysis kits with ELISA: determinations of a panel of nine cytokines in clinical sample culture supernatants*. J Reprod Immun*ol 2005**;** 66(**2)**: 175-191.
